# Supplementary material for: Designing and interpreting 4D tumour spheroid experiments
Source: Commun Biol. 2022 Jan 24;5:91. doi: 10.1038/s42003-022-03018-3 (PMC8786869; doi:10.1038/s42003-022-03018-3)
Supplement: Supplementary file 3 — Description of Additional Supplementary Files [file 42003_2022_3018_MOESM3_ESM.pdf]

Description of Additional Supplementary Files

File name: Supplementary Data 1
Description: Data associated with the Figures in the main manuscript.
