# Supplementary material for: Designing and interpreting 4D tumour spheroid experiments
Source: Commun Biol. 2022 Jan 24;5:91. doi: 10.1038/s42003-022-03018-3 (PMC8786869; doi:10.1038/s42003-022-03018-3)
Supplement: Supplementary file 5 — Reporting Summary [file 42003_2022_3018_MOESM5_ESM.pdf]

nature research

Corresponding author(s): Matthew J. Simpson

Last updated by author(s): Dec 23, 2021

Reporting Summary

Nature Research wishes to improve the reproducibility of the work that we publish. This form provides structure for consistency and transparency in reporting. For further information on Nature Research policies, see our Editorial Policies and the Editorial Policy Checklist.

Statistics

For all statistical analyses, confirm that the following items are present in the figure legend, table legend, main text, or Methods section.

n/a	Confirmed	
☐	☒	The exact sample size (n) for each experimental group/condition, given as a discrete number and unit of measurement
☐	☒	A statement on whether measurements were taken from distinct samples or whether the same sample was measured repeatedly
☒	☐	The statistical test(s) used AND whether they are one- or two-sided<br>*Only common tests should be described solely by name; describe more complex techniques in the Methods section.*
☒	☐	A description of all covariates tested
☒	☐	A description of any assumptions or corrections, such as tests of normality and adjustment for multiple comparisons
☒	☐	A full description of the statistical parameters including central tendency (e.g. means) or other basic estimates (e.g. regression coefficient) AND variation (e.g. standard deviation) or associated estimates of uncertainty (e.g. confidence intervals)
☒	☐	For null hypothesis testing, the test statistic (e.g. F, t, r) with confidence intervals, effect sizes, degrees of freedom and P value noted<br>*Give P values as exact values whenever suitable.*
☒	☐	For Bayesian analysis, information on the choice of priors and Markov chain Monte Carlo settings
☒	☐	For hierarchical and complex designs, identification of the appropriate level for tests and full reporting of outcomes
☒	☐	Estimates of effect sizes (e.g. Cohen's d, Pearson's r), indicating how they were calculated

Our web collection on statistics for biologists contains articles on many of the points above.

Software and code

Policy information about availability of computer code

Data collection	IncuCyte S3 live cell imaging system (Sartorius, Goettingen, Germany) was used to capture images of one 96-well plate of spheroids per cell line. Other spheroid images were captured using an Olympus FV3000 confocal microscope. Custom-code written in MATLAB (v2021) was used to simulate the mathematical model and perform statistical identifiability analysis.
Data analysis	IncuCyte S3 2020C Rev1 software was used to analyse images of spheroids obtained in the IncuCyte S3 live cell imaging system (Sartorius, Goettingen, Germany) to obtain outer radius measurements of one 96-well plate of spheroids per cell line. Other outer radius measurements, inhibited and necrotic core measurements were obtained by image processing by first converting confocal microscopy images to TIFF files in ImageJ and then processed with custom MATLAB scripts that use standard MATLAB image processing toolbox functions. These scripts are freely available on Zenodo with DOI:10.5281/zenodo.5121093. Custom-code written in MATLAB (v2021) was used to simulate the mathematical model and perform statistical identifiability analysis using functions from the Optimization Toolbox, Global Optimization Toolbox and the Statistics and Machine Learning Toolbox. The custom-code is available on a GitHub repository on a GitHub repository (https://github.com/ryanmurphy42/4DSpheroids_Murphy2021).

For manuscripts utilizing custom algorithms or software that are central to the research but not yet described in published literature, software must be made available to editors and reviewers. We strongly encourage code deposition in a community repository (e.g. GitHub). See the Nature Research guidelines for submitting code & software for further information.

Data

Policy information about availability of data

All manuscripts must include a data availability statement. This statement should provide the following information, where applicable:

- Accession codes, unique identifiers, or web links for publicly available datasets
- A list of figures that have associated raw data
- A description of any restrictions on data availability

The datasets generated during and analysed during the current study are available on a GitHub repository (https://github.com/ryanmurphy42/4DSpheroids_Murphy2021) and are summarised in the electronic supplementary material. In addition, Supplementary Data 1 contains the data presented in the figures of the main manuscript, focusing on the WM793b cell line.

Field-specific reporting

Please select the one below that is the best fit for your research. If you are not sure, read the appropriate sections before making your selection.

☒ Life sciences ☐ Behavioural & social sciences ☐ Ecological, evolutionary & environmental sciences

For a reference copy of the document with all sections, see nature.com/documents/nr-reporting-summary-flat.pdf

Life sciences study design

All studies must disclose on these points even when the disclosure is negative.

Sample size	For in vitro experiments, no sample size calculation was performed prior to experiment. However, part of this study is to compare the information gained using different experimental designs, for example temporal resolutions, and by comparing these results we determine when our sample size is sufficient.
Data exclusions	Some measurements could not be obtained primarily due to blurring of the automated imaging, spheroids not forming properly, or spheroids losing their structural integrity at very late time. Data for these spheroids were excluded.
Replication	Three different cell lines and multiple plates thereof were seeded with spheroids at the initial time point. Time series data presented in the paper and comparison of results across multiple experimental designs demonstrate the reproducibility of experimental findings.
Randomization	For in vitro experiments, randomization was not possible. However, experiments were set up so that after multiple plates were seeded to form spheroids at the initial time point, spheroids were randomly selected at each data collection time point to harvest and obtain outer, inhibited, and necrotic radii measurements.
Blinding	For in vitro experiments, blinding was not possible for data collection. However, image analysis was semi-automated and mathematical modelling and statistical identifiability analysis was automated to minimize bias.

Reporting for specific materials, systems and methods

We require information from authors about some types of materials, experimental systems and methods used in many studies. Here, indicate whether each material, system or method listed is relevant to your study. If you are not sure if a list item applies to your research, read the appropriate section before selecting a response.

Materials & experimental systems

n/a	Involved in the study	
☒	☐	Antibodies
☐	☒	Eukaryotic cell lines
☒	☐	Palaeontology and archaeology
☒	☐	Animals and other organisms
☒	☐	Human research participants
☒	☐	Clinical data
☒	☐	Dual use research of concern

Methods

n/a	Involved in the study	
☒	☐	ChIP-seq
☒	☐	Flow cytometry
☒	☐	MRI-based neuroimaging

Eukaryotic cell lines

Policy information about cell lines

Cell line source(s)	The human melanoma cell lines WM793b, WM983b, and WM164 were sourced from The Wistar Institute, Philadelphia, PA.

Authentication	The human melanoma cell lines WM793b, WM983b, and WM164 were authenticated by short tandem repeat fingerprinting (QIMR Berghofer Medical Research Institute, Herston, Australia).
Mycoplasma contamination	Cell lines were checked routinely for mycoplasma and tested negative using the MycoAlert MycoPlasma Detection Kit (Lonza) and PCR (Uphoff & Drexler, Methods in Molecular Biology 2011, 731, 93).
Commonly misidentified lines (See ICLAC register)	None
